# (2-Bromo­acet­yl)ferrocene

**DOI:** 10.1107/S1600536811050434

**Published:** 2011-11-30

**Authors:** Xiang-Xiang Wu, Xin Zhu, Qiu-Juan Ma, Seik Weng Ng, Edward R. T. Tiekink

**Affiliations:** a Henan University of Traditional Medicine, Zhengzhou 450008, People’s Republic of China; b Department of Chemistry, University of Malaya, 50603 Kuala Lumpur, Malaysia; c Chemistry Department, Faculty of Science, King Abdulaziz University, PO Box 80203 Jeddah, Saudi Arabia

## Abstract

In the title mol­ecule, [Fe(C_5_H_5_)(C_7_H_6_BrO)], the C atoms of the substituted ring have disparate Fe—C bond lengths compared with the unsubstituted ring. In the bromo­acetyl residue, the Br and O atoms are co-planar [the O—C—C—Br torsion angle is 5.7 (4)°] and are *syn* to each other. Helical supra­molecular chains along the *b* axis are formed in the crystal structure mediated by C—H⋯O contacts; the carbonyl-O atom is bifurcated. The chains are linked into layers by C—H⋯π(unsubstituted ring) inter­actions that stack along the *a*-axis direction.

## Related literature

For background to the potential applications of ferrocenyl derivatives in medicine and as biosensors, see: Arezki *et al.* (2011 ▶); Huang *et al.* (2008 ▶); Yang *et al.* (2007 ▶).
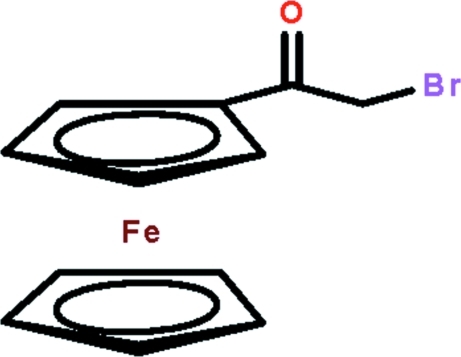


## Experimental

### 

#### Crystal data

[Fe(C_5_H_5_)(C_7_H_6_BrO)]
*M* *_r_* = 306.97
Monoclinic, 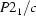

*a* = 7.7095 (3) Å
*b* = 9.6609 (4) Å
*c* = 14.7464 (7) Å
β = 98.061 (4)°
*V* = 1087.47 (8) Å^3^
*Z* = 4
Mo *K*α radiation
μ = 5.03 mm^−1^
*T* = 100 K
0.30 × 0.10 × 0.03 mm

#### Data collection

Agilent SuperNova Dual diffractometer with Atlas detector
Absorption correction: multi-scan (*CrysAlis PRO*; Agilent, 2010 ▶) *T*_min_ = 0.314, *T*_max_ = 0.864
7692 measured reflections
2497 independent reflections
2004 reflections with *I* > 2σ(*I*)
*R*_int_ = 0.045

#### Refinement

*R*[*F* ^2^ > 2σ(*F* ^2^)] = 0.035
*wR*(*F* ^2^) = 0.079
*S* = 1.02
2497 reflections
136 parameters
H-atom parameters constrained
Δρ_max_ = 0.73 e Å^−3^
Δρ_min_ = −0.58 e Å^−3^

### 

Data collection: *CrysAlis PRO* (Agilent, 2010 ▶); cell refinement: *CrysAlis PRO*; data reduction: *CrysAlis PRO*; program(s) used to solve structure: *SHELXS97* (Sheldrick, 2008 ▶); program(s) used to refine structure: *SHELXL97* (Sheldrick, 2008 ▶); molecular graphics: *X-SEED* (Barbour, 2001 ▶) and *DIAMOND* (Brandenburg, 2006 ▶); software used to prepare material for publication: *PLATON* (Spek, 2009 ▶) and *publCIF* (Westrip, 2010 ▶).

## Supplementary Material

Crystal structure: contains datablock(s) global, I. DOI: 10.1107/S1600536811050434/hg5144sup1.cif

Structure factors: contains datablock(s) I. DOI: 10.1107/S1600536811050434/hg5144Isup2.hkl

Additional supplementary materials:  crystallographic information; 3D view; checkCIF report

## Figures and Tables

**Table 1:** Selected bond lengths (Å)

Fe1—C1	2.053 (3)
Fe1—C2	2.051 (3)
Fe1—C3	2.042 (3)
Fe1—C4	2.040 (3)
Fe1—C5	2.040 (3)
Fe1—C6	2.037 (3)
Fe1—C7	2.060 (3)
Fe1—C8	2.059 (3)
Fe1—C9	2.043 (3)
Fe1—C10	2.027 (3)

**Table 2:** Hydrogen-bond geometry (Å, °) *Cg*1 is the centroid of the C1–C5 ring.

*D*—H⋯*A*	*D*—H	H⋯*A*	*D*⋯*A*	*D*—H⋯*A*
C1—H1⋯O1^i^	1.00	2.49	3.327 (4)	140
C12—H12a⋯O1^i^	0.99	2.35	3.291 (4)	158
C12—H12b⋯*Cg*1^ii^	0.99	2.64	3.445 (3)	139

Symmetry codes: (i) 
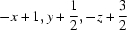
; (ii) 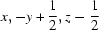.

**Fig. 1.**
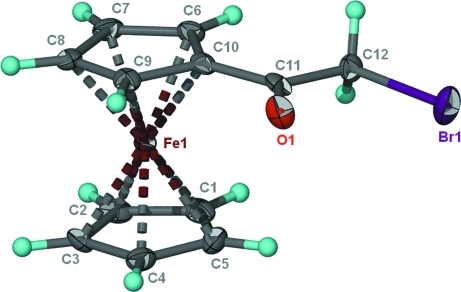
Molecular structure of (I) showing atom-labelling scheme and displacement ellipsoids at the 70% probability level.

**Fig. 2.**
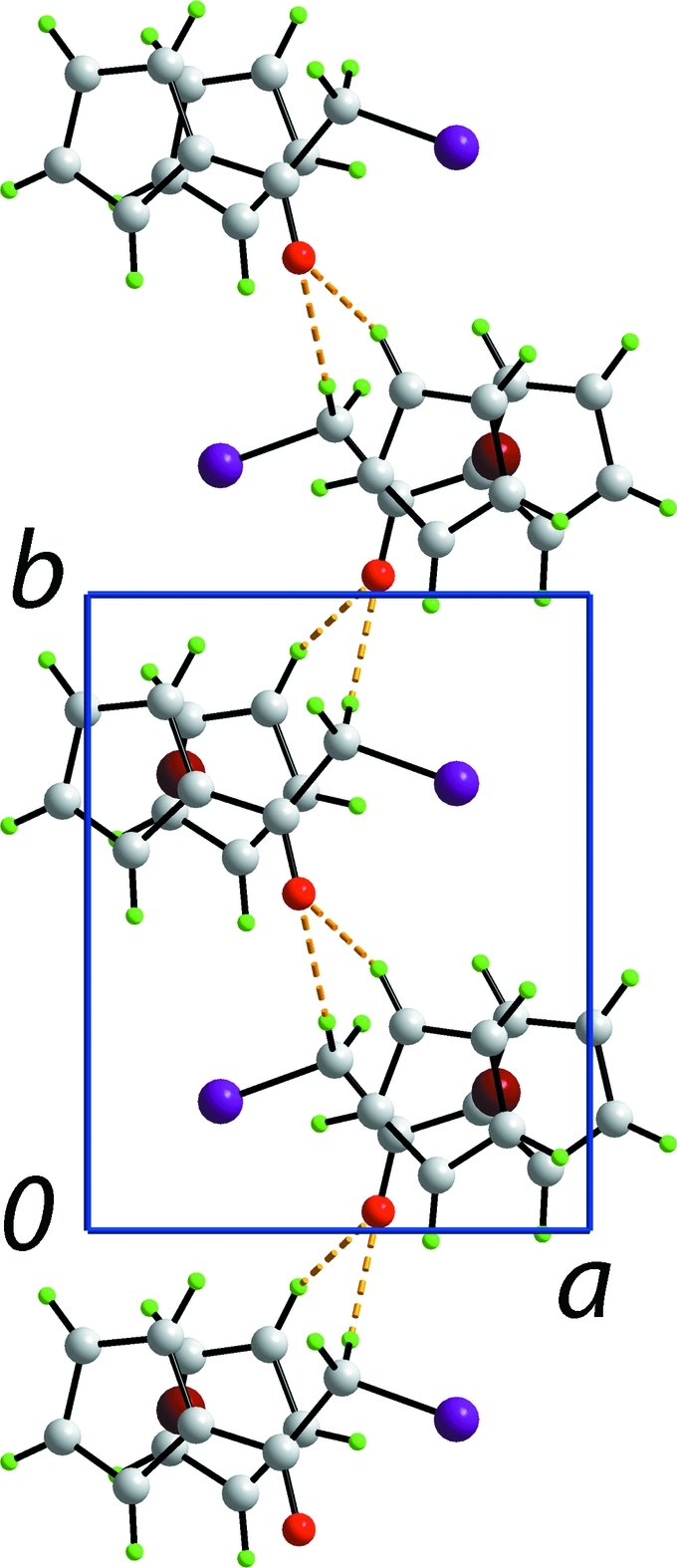
Supramolecular helical chain along the b axis in (I). The C—H···O contacts are shown as orange dashed lines.

**Fig. 3.**
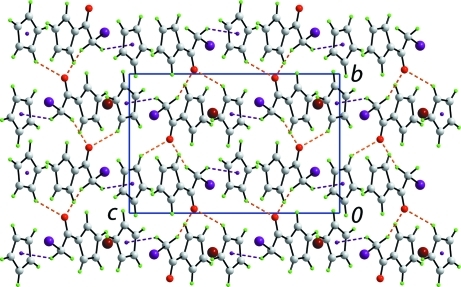
Supramolecular layer in the bc plane in (I). The C—H···O and C—H···π contacts are shown as orange and purple dashed lines, respectively.

**Fig. 4.**
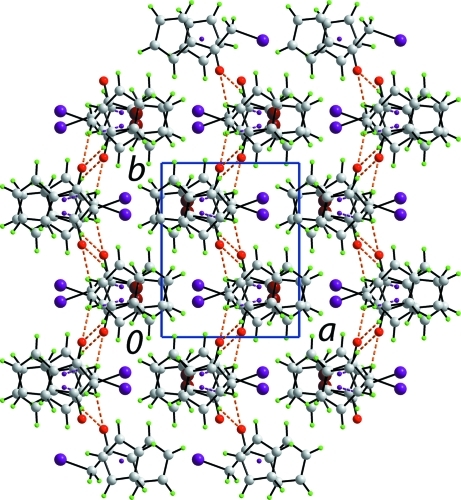
Stacking of layers along the a axis in (I). The C—H···O and C—H···π contacts are shown as orange and purple dashed lines, respectively.

## supplementary crystallographic information

### Comment

Ferrocenyl derivatives continue to attract attention owing to their applications as biosensors and drugs (Arezki *et al.*, 2011; Huang *et al.*, 2008; Yang *et al.*, 2007). The title compound, (I), is a synthetic precursor for such molecules.

The molecular structure of (I), Fig. 1, features substituted and unsubstituted cyclopentadienyl rings which form a dihedral angle of 1.14 (18)° indicating an almost parallel relationship; the rings are almost eclipsed when viewed down the ring centroid···Fe···ring centroid axis. The Fe—C bond distances formed by the C atoms in the unsubstituted ring are equal within experimental error, *i.e*. 2.040 (3) to 2.053 (3) Å, but a disparity is evident in the Fe—C bond distances formed by the substituted ring, range of Fe—C is 2.027 (3) Å, for the C atom carrying the substituent, to 2.060 (3) Å; Table 1. The average Fe—C bond distances are experimentally equivalent as is reflected in the Fe···ring centroid distances of 1.6480 (14), for the C1-ring, compared to 1.6447 (14) Å for the C6-ring; the ring centroid···Fe···ring centroid angle is 179.68 (7)°. In the bromoacetyl residue, the Br and O atoms are co-planar [the O1—C11—C12—Br1 torsion angle is 5.7 (4)°] and are *syn* to each other.

Supramolecular helical chains along the *b* axis and mediated by C—H···O contacts involving the bifurcated carbonyl-O atom feature in the crystal packing, Fig. 2. These are connected into layers in the *bc* plane by C—H···π contacts, Fig. 3. The layers are undulating and stack along the *a* axis, Fig. 4.

### Experimental

A flask was charged with ferrocene (281 mg, 1.51 mmol) and 2-bromoacetyl bromide (305 mg, 1.51 mmol) in dichloromethane (30 ml); the flask was chilled in an ice-bath. To the solution was added anhydrous aluminium chloride (221 mg, 1.66 mmol) under a nitrogen atmosphere. The reaction mixture, which turned deep-violet, was stirred for an hour. Water was added and the organic phase was extracted with dichloromethane (3 × 15 ml). The organic layer was dried over anhydrous sodium sulfate. The solvent was removed and the product purified by flash column chromatography (ethyl acetate/hexane, 1:9) to give the product as a brown solid; crystals were grown from its solution in dichloromethane.

### Refinement

Carbon-bound H-atoms were placed in calculated positions [C—H 0.99 to 1.00 Å, *U*_iso_(H) 1.2*U*_eq_(C)] and were included in the refinement in the riding model approximation.

### Crystal data

**Table:**

[Fe(C_5_H_5_)(C_7_H_6_BrO)]	*F*(000) = 608
*M**_r_* = 306.97	*D*_x_ = 1.875 Mg m^−^^3^
Monoclinic, *P*2_1_/*c*	Mo *K*α radiation, λ = 0.71073 Å
Hall symbol: -P 2ybc	Cell parameters from 2673 reflections
*a* = 7.7095 (3) Å	θ = 2.5–27.5°
*b* = 9.6609 (4) Å	µ = 5.03 mm^−^^1^
*c* = 14.7464 (7) Å	*T* = 100 K
β = 98.061 (4)°	Plate, brown
*V* = 1087.47 (8) Å^3^	0.30 × 0.10 × 0.03 mm
*Z* = 4	

### Data collection

**Table:**

Agilent SuperNova Dual diffractometer with Atlas detector	2497 independent reflections
Radiation source: SuperNova (Mo) X-ray Source	2004 reflections with *I* > 2σ(*I*)
Mirror	*R*_int_ = 0.045
Detector resolution: 10.4041 pixels mm^-1^	θ_max_ = 27.6°, θ_min_ = 2.5°
ω scan	*h* = −7→10
Absorption correction: multi-scan (*CrysAlis PRO*; Agilent, 2010)	*k* = −12→12
*T*_min_ = 0.314, *T*_max_ = 0.864	*l* = −19→19
7692 measured reflections	

### Refinement

**Table:**

Refinement on *F*^2^	Primary atom site location: structure-invariant direct methods
Least-squares matrix: full	Secondary atom site location: difference Fourier map
*R*[*F*^2^ > 2σ(*F*^2^)] = 0.035	Hydrogen site location: inferred from neighbouring sites
*wR*(*F*^2^) = 0.079	H-atom parameters constrained
*S* = 1.02	*w* = 1/[σ^2^(*F*_o_^2^) + (0.0276*P*)^2^ + 0.4313*P*] where *P* = (*F*_o_^2^ + 2*F*_c_^2^)/3
2497 reflections	(Δ/σ)_max_ = 0.001
136 parameters	Δρ_max_ = 0.73 e Å^−^^3^
0 restraints	Δρ_min_ = −0.58 e Å^−^^3^

### Fractional atomic coordinates and isotropic or equivalent isotropic displacement parameters (Å^2^)

**Table:**

	*x*	*y*	*z*	*U*_iso_*/*U*_eq_	
Br1	0.73500 (4)	0.70286 (3)	0.88244 (3)	0.02647 (12)	
Fe1	0.18625 (5)	0.71721 (4)	0.60388 (3)	0.01114 (12)	
O1	0.4210 (3)	0.5296 (2)	0.80778 (15)	0.0200 (5)	
C1	0.3624 (4)	0.8207 (3)	0.5363 (2)	0.0201 (7)	
H1	0.4199	0.9111	0.5548	0.024*	
C2	0.2000 (4)	0.8024 (3)	0.4777 (2)	0.0186 (7)	
H2	0.1235	0.8781	0.4483	0.022*	
C3	0.1643 (4)	0.6583 (3)	0.4698 (2)	0.0160 (6)	
H3	0.0591	0.6149	0.4333	0.019*	
C4	0.3048 (4)	0.5867 (3)	0.5228 (2)	0.0174 (7)	
H4	0.3161	0.4840	0.5301	0.021*	
C5	0.4263 (4)	0.6876 (3)	0.5640 (2)	0.0218 (7)	
H5	0.5375	0.6678	0.6055	0.026*	
C6	0.1556 (4)	0.8318 (3)	0.7165 (2)	0.0154 (6)	
H6	0.2173	0.9202	0.7359	0.019*	
C7	−0.0085 (4)	0.8188 (3)	0.6598 (2)	0.0191 (7)	
H7	−0.0807	0.8971	0.6307	0.023*	
C8	−0.0498 (4)	0.6754 (3)	0.6484 (2)	0.0181 (7)	
H8	−0.1560	0.6362	0.6103	0.022*	
C9	0.0873 (4)	0.5979 (3)	0.6989 (2)	0.0155 (6)	
H9	0.0938	0.4947	0.7034	0.019*	
C10	0.2161 (4)	0.6937 (3)	0.7418 (2)	0.0140 (6)	
C11	0.3856 (4)	0.6510 (3)	0.7928 (2)	0.0145 (6)	
C12	0.5074 (4)	0.7682 (3)	0.8252 (2)	0.0161 (7)	
H12B	0.4538	0.8252	0.8697	0.019*	
H12A	0.5232	0.8278	0.7723	0.019*	

### Atomic displacement parameters (Å^2^)

**Table:**

	*U*^11^	*U*^22^	*U*^33^	*U*^12^	*U*^13^	*U*^23^
Br1	0.01576 (18)	0.0226 (2)	0.0391 (2)	0.00046 (12)	−0.00282 (16)	−0.00281 (15)
Fe1	0.0115 (2)	0.0123 (2)	0.0098 (2)	0.00045 (16)	0.00201 (18)	−0.00059 (17)
O1	0.0241 (12)	0.0117 (12)	0.0221 (13)	0.0019 (9)	−0.0036 (10)	−0.0007 (9)
C1	0.0239 (17)	0.0225 (17)	0.0161 (16)	−0.0087 (14)	0.0102 (15)	−0.0031 (14)
C2	0.0256 (18)	0.0183 (17)	0.0128 (16)	0.0034 (13)	0.0063 (14)	0.0024 (13)
C3	0.0206 (16)	0.0173 (16)	0.0098 (15)	0.0000 (13)	0.0005 (13)	−0.0041 (13)
C4	0.0176 (15)	0.0181 (16)	0.0170 (16)	0.0055 (12)	0.0040 (14)	−0.0004 (13)
C5	0.0124 (15)	0.036 (2)	0.0177 (17)	0.0007 (14)	0.0029 (14)	−0.0019 (15)
C6	0.0184 (16)	0.0116 (15)	0.0165 (16)	0.0019 (12)	0.0034 (14)	−0.0038 (13)
C7	0.0183 (16)	0.0231 (17)	0.0163 (16)	0.0099 (13)	0.0036 (14)	−0.0005 (14)
C8	0.0130 (15)	0.0247 (17)	0.0178 (17)	−0.0023 (13)	0.0066 (14)	−0.0057 (14)
C9	0.0175 (15)	0.0178 (16)	0.0122 (15)	−0.0033 (12)	0.0056 (13)	0.0008 (13)
C10	0.0173 (15)	0.0170 (16)	0.0080 (14)	−0.0014 (12)	0.0031 (13)	−0.0001 (12)
C11	0.0208 (16)	0.0155 (16)	0.0071 (14)	−0.0013 (13)	0.0019 (13)	−0.0016 (12)
C12	0.0138 (15)	0.0175 (16)	0.0160 (16)	0.0025 (12)	−0.0020 (13)	0.0000 (13)

### Geometric parameters (Å, °)

**Table:**

Br1—C12	1.943 (3)	C3—H3	1.0000
Fe1—C1	2.053 (3)	C4—C5	1.428 (4)
Fe1—C2	2.051 (3)	C4—H4	1.0000
Fe1—C3	2.042 (3)	C5—H5	1.0000
Fe1—C4	2.040 (3)	C6—C7	1.421 (5)
Fe1—C5	2.040 (3)	C6—C10	1.445 (4)
Fe1—C6	2.037 (3)	C6—H6	1.0000
Fe1—C7	2.060 (3)	C7—C8	1.426 (4)
Fe1—C8	2.059 (3)	C7—H7	1.0000
Fe1—C9	2.043 (3)	C8—C9	1.418 (4)
Fe1—C10	2.027 (3)	C8—H8	1.0000
O1—C11	1.217 (3)	C9—C10	1.438 (4)
C1—C5	1.416 (4)	C9—H9	1.0000
C1—C2	1.429 (5)	C10—C11	1.473 (4)
C1—H1	1.0000	C11—C12	1.506 (4)
C2—C3	1.421 (4)	C12—H12B	0.9900
C2—H2	1.0000	C12—H12A	0.9900
C3—C4	1.423 (4)		
			
C10—Fe1—C6	41.65 (12)	C4—C3—C2	107.9 (3)
C10—Fe1—C4	121.43 (12)	C4—C3—Fe1	69.53 (17)
C6—Fe1—C4	157.89 (13)	C2—C3—Fe1	70.02 (17)
C10—Fe1—C5	107.09 (13)	C4—C3—H3	126.1
C6—Fe1—C5	121.68 (13)	C2—C3—H3	126.1
C4—Fe1—C5	40.97 (12)	Fe1—C3—H3	126.1
C10—Fe1—C3	157.32 (12)	C3—C4—C5	107.8 (3)
C6—Fe1—C3	159.69 (12)	C3—C4—Fe1	69.65 (16)
C4—Fe1—C3	40.82 (12)	C5—C4—Fe1	69.50 (17)
C5—Fe1—C3	68.70 (13)	C3—C4—H4	126.1
C10—Fe1—C9	41.37 (12)	C5—C4—H4	126.1
C6—Fe1—C9	69.52 (12)	Fe1—C4—H4	126.1
C4—Fe1—C9	107.11 (12)	C1—C5—C4	108.4 (3)
C5—Fe1—C9	124.08 (13)	C1—C5—Fe1	70.27 (18)
C3—Fe1—C9	121.24 (12)	C4—C5—Fe1	69.53 (17)
C10—Fe1—C2	160.42 (12)	C1—C5—H5	125.8
C6—Fe1—C2	123.26 (12)	C4—C5—H5	125.8
C4—Fe1—C2	68.40 (12)	Fe1—C5—H5	125.8
C5—Fe1—C2	68.27 (13)	C7—C6—C10	107.4 (3)
C3—Fe1—C2	40.63 (11)	C7—C6—Fe1	70.56 (18)
C9—Fe1—C2	156.99 (13)	C10—C6—Fe1	68.79 (16)
C10—Fe1—C1	123.48 (13)	C7—C6—H6	126.3
C6—Fe1—C1	106.94 (13)	C10—C6—H6	126.3
C4—Fe1—C1	68.62 (13)	Fe1—C6—H6	126.3
C5—Fe1—C1	40.49 (13)	C6—C7—C8	108.7 (3)
C3—Fe1—C1	68.68 (13)	C6—C7—Fe1	68.85 (17)
C9—Fe1—C1	160.67 (13)	C8—C7—Fe1	69.73 (16)
C2—Fe1—C1	40.75 (13)	C6—C7—H7	125.6
C10—Fe1—C7	68.82 (12)	C8—C7—H7	125.6
C6—Fe1—C7	40.58 (13)	Fe1—C7—H7	125.6
C4—Fe1—C7	160.14 (13)	C9—C8—C7	108.2 (3)
C5—Fe1—C7	157.53 (13)	C9—C8—Fe1	69.15 (16)
C3—Fe1—C7	123.65 (13)	C7—C8—Fe1	69.75 (17)
C9—Fe1—C7	68.35 (12)	C9—C8—H8	125.9
C2—Fe1—C7	107.77 (13)	C7—C8—H8	125.9
C1—Fe1—C7	122.06 (13)	Fe1—C8—H8	125.9
C10—Fe1—C8	68.87 (13)	C8—C9—C10	108.0 (3)
C6—Fe1—C8	68.78 (12)	C8—C9—Fe1	70.40 (17)
C4—Fe1—C8	123.64 (12)	C10—C9—Fe1	68.70 (16)
C5—Fe1—C8	160.53 (13)	C8—C9—H9	126.0
C3—Fe1—C8	107.26 (13)	C10—C9—H9	126.0
C9—Fe1—C8	40.45 (12)	Fe1—C9—H9	126.0
C2—Fe1—C8	121.89 (14)	C9—C10—C6	107.6 (3)
C1—Fe1—C8	157.56 (13)	C9—C10—C11	123.6 (3)
C7—Fe1—C8	40.52 (12)	C6—C10—C11	128.4 (3)
C5—C1—C2	107.6 (3)	C9—C10—Fe1	69.93 (17)
C5—C1—Fe1	69.25 (17)	C6—C10—Fe1	69.57 (17)
C2—C1—Fe1	69.53 (17)	C11—C10—Fe1	120.8 (2)
C5—C1—H1	126.2	O1—C11—C10	121.5 (3)
C2—C1—H1	126.2	O1—C11—C12	123.6 (3)
Fe1—C1—H1	126.2	C10—C11—C12	114.9 (3)
C3—C2—C1	108.3 (3)	C11—C12—Br1	112.2 (2)
C3—C2—Fe1	69.35 (17)	C11—C12—H12B	109.2
C1—C2—Fe1	69.72 (18)	Br1—C12—H12B	109.2
C3—C2—H2	125.8	C11—C12—H12A	109.2
C1—C2—H2	125.8	Br1—C12—H12A	109.2
Fe1—C2—H2	125.8	H12B—C12—H12A	107.9
			
C10—Fe1—C1—C5	76.6 (2)	C9—Fe1—C6—C10	38.23 (17)
C6—Fe1—C1—C5	119.3 (2)	C2—Fe1—C6—C10	−163.33 (18)
C4—Fe1—C1—C5	−37.78 (19)	C1—Fe1—C6—C10	−121.69 (19)
C3—Fe1—C1—C5	−81.8 (2)	C7—Fe1—C6—C10	118.5 (3)
C9—Fe1—C1—C5	42.9 (5)	C8—Fe1—C6—C10	81.65 (19)
C2—Fe1—C1—C5	−119.1 (3)	C10—C6—C7—C8	−0.7 (3)
C7—Fe1—C1—C5	161.02 (19)	Fe1—C6—C7—C8	58.4 (2)
C8—Fe1—C1—C5	−165.4 (3)	C10—C6—C7—Fe1	−59.2 (2)
C10—Fe1—C1—C2	−164.34 (17)	C10—Fe1—C7—C6	38.79 (18)
C6—Fe1—C1—C2	−121.66 (19)	C4—Fe1—C7—C6	163.8 (3)
C4—Fe1—C1—C2	81.30 (19)	C5—Fe1—C7—C6	−44.8 (4)
C5—Fe1—C1—C2	119.1 (3)	C3—Fe1—C7—C6	−162.66 (18)
C3—Fe1—C1—C2	37.33 (17)	C9—Fe1—C7—C6	83.4 (2)
C9—Fe1—C1—C2	162.0 (3)	C2—Fe1—C7—C6	−120.74 (19)
C7—Fe1—C1—C2	−79.9 (2)	C1—Fe1—C7—C6	−78.3 (2)
C8—Fe1—C1—C2	−46.3 (4)	C8—Fe1—C7—C6	120.6 (3)
C5—C1—C2—C3	0.2 (4)	C10—Fe1—C7—C8	−81.9 (2)
Fe1—C1—C2—C3	−58.8 (2)	C6—Fe1—C7—C8	−120.6 (3)
C5—C1—C2—Fe1	59.0 (2)	C4—Fe1—C7—C8	43.1 (5)
C10—Fe1—C2—C3	162.0 (3)	C5—Fe1—C7—C8	−165.4 (3)
C6—Fe1—C2—C3	−163.31 (17)	C3—Fe1—C7—C8	76.7 (2)
C4—Fe1—C2—C3	37.94 (18)	C9—Fe1—C7—C8	−37.27 (19)
C5—Fe1—C2—C3	82.18 (19)	C2—Fe1—C7—C8	118.6 (2)
C9—Fe1—C2—C3	−45.0 (4)	C1—Fe1—C7—C8	161.04 (19)
C1—Fe1—C2—C3	119.8 (3)	C6—C7—C8—C9	0.7 (3)
C7—Fe1—C2—C3	−121.34 (19)	Fe1—C7—C8—C9	58.6 (2)
C8—Fe1—C2—C3	−79.1 (2)	C6—C7—C8—Fe1	−57.9 (2)
C10—Fe1—C2—C1	42.2 (4)	C10—Fe1—C8—C9	−38.11 (18)
C6—Fe1—C2—C1	76.9 (2)	C6—Fe1—C8—C9	−82.9 (2)
C4—Fe1—C2—C1	−81.9 (2)	C4—Fe1—C8—C9	76.4 (2)
C5—Fe1—C2—C1	−37.65 (18)	C5—Fe1—C8—C9	43.4 (5)
C3—Fe1—C2—C1	−119.8 (3)	C3—Fe1—C8—C9	118.20 (19)
C9—Fe1—C2—C1	−164.8 (3)	C2—Fe1—C8—C9	160.24 (18)
C7—Fe1—C2—C1	118.8 (2)	C1—Fe1—C8—C9	−166.0 (3)
C8—Fe1—C2—C1	161.02 (18)	C7—Fe1—C8—C9	−119.8 (3)
C1—C2—C3—C4	−0.4 (3)	C10—Fe1—C8—C7	81.7 (2)
Fe1—C2—C3—C4	−59.4 (2)	C6—Fe1—C8—C7	36.90 (19)
C1—C2—C3—Fe1	59.0 (2)	C4—Fe1—C8—C7	−163.81 (19)
C10—Fe1—C3—C4	−45.4 (4)	C5—Fe1—C8—C7	163.2 (4)
C6—Fe1—C3—C4	162.8 (3)	C3—Fe1—C8—C7	−121.98 (19)
C5—Fe1—C3—C4	37.96 (18)	C9—Fe1—C8—C7	119.8 (3)
C9—Fe1—C3—C4	−79.9 (2)	C2—Fe1—C8—C7	−79.9 (2)
C2—Fe1—C3—C4	119.0 (3)	C1—Fe1—C8—C7	−46.2 (4)
C1—Fe1—C3—C4	81.6 (2)	C7—C8—C9—C10	−0.4 (3)
C7—Fe1—C3—C4	−163.30 (18)	Fe1—C8—C9—C10	58.58 (19)
C8—Fe1—C3—C4	−121.84 (18)	C7—C8—C9—Fe1	−59.0 (2)
C10—Fe1—C3—C2	−164.4 (3)	C10—Fe1—C9—C8	119.4 (3)
C6—Fe1—C3—C2	43.8 (4)	C6—Fe1—C9—C8	80.94 (19)
C4—Fe1—C3—C2	−119.0 (3)	C4—Fe1—C9—C8	−122.16 (19)
C5—Fe1—C3—C2	−81.0 (2)	C5—Fe1—C9—C8	−163.95 (19)
C9—Fe1—C3—C2	161.13 (19)	C3—Fe1—C9—C8	−79.8 (2)
C1—Fe1—C3—C2	−37.43 (19)	C2—Fe1—C9—C8	−47.3 (4)
C7—Fe1—C3—C2	77.7 (2)	C1—Fe1—C9—C8	163.8 (3)
C8—Fe1—C3—C2	119.16 (19)	C7—Fe1—C9—C8	37.33 (18)
C2—C3—C4—C5	0.5 (3)	C6—Fe1—C9—C10	−38.48 (17)
Fe1—C3—C4—C5	−59.3 (2)	C4—Fe1—C9—C10	118.42 (18)
C2—C3—C4—Fe1	59.7 (2)	C5—Fe1—C9—C10	76.6 (2)
C10—Fe1—C4—C3	161.21 (17)	C3—Fe1—C9—C10	160.74 (17)
C6—Fe1—C4—C3	−164.1 (3)	C2—Fe1—C9—C10	−166.7 (3)
C5—Fe1—C4—C3	−119.1 (3)	C1—Fe1—C9—C10	44.4 (4)
C9—Fe1—C4—C3	118.28 (18)	C7—Fe1—C9—C10	−82.09 (19)
C2—Fe1—C4—C3	−37.77 (18)	C8—Fe1—C9—C10	−119.4 (3)
C1—Fe1—C4—C3	−81.7 (2)	C8—C9—C10—C6	0.0 (3)
C7—Fe1—C4—C3	44.8 (4)	Fe1—C9—C10—C6	59.60 (19)
C8—Fe1—C4—C3	77.0 (2)	C8—C9—C10—C11	−174.0 (3)
C10—Fe1—C4—C5	−79.7 (2)	Fe1—C9—C10—C11	−114.3 (3)
C6—Fe1—C4—C5	−45.1 (4)	C8—C9—C10—Fe1	−59.6 (2)
C3—Fe1—C4—C5	119.1 (3)	C7—C6—C10—C9	0.5 (3)
C9—Fe1—C4—C5	−122.7 (2)	Fe1—C6—C10—C9	−59.82 (19)
C2—Fe1—C4—C5	81.3 (2)	C7—C6—C10—C11	174.0 (3)
C1—Fe1—C4—C5	37.35 (19)	Fe1—C6—C10—C11	113.7 (3)
C7—Fe1—C4—C5	163.8 (3)	C7—C6—C10—Fe1	60.3 (2)
C8—Fe1—C4—C5	−163.94 (19)	C6—Fe1—C10—C9	118.7 (2)
C2—C1—C5—C4	0.1 (3)	C4—Fe1—C10—C9	−80.1 (2)
Fe1—C1—C5—C4	59.2 (2)	C5—Fe1—C10—C9	−122.53 (18)
C2—C1—C5—Fe1	−59.2 (2)	C3—Fe1—C10—C9	−47.0 (4)
C3—C4—C5—C1	−0.3 (3)	C2—Fe1—C10—C9	164.4 (3)
Fe1—C4—C5—C1	−59.7 (2)	C1—Fe1—C10—C9	−163.89 (18)
C3—C4—C5—Fe1	59.4 (2)	C7—Fe1—C10—C9	80.88 (19)
C10—Fe1—C5—C1	−121.9 (2)	C8—Fe1—C10—C9	37.29 (17)
C6—Fe1—C5—C1	−78.7 (2)	C4—Fe1—C10—C6	161.21 (18)
C4—Fe1—C5—C1	119.5 (3)	C5—Fe1—C10—C6	118.76 (19)
C3—Fe1—C5—C1	81.7 (2)	C3—Fe1—C10—C6	−165.7 (3)
C9—Fe1—C5—C1	−164.20 (19)	C9—Fe1—C10—C6	−118.7 (2)
C2—Fe1—C5—C1	37.88 (19)	C2—Fe1—C10—C6	45.7 (4)
C7—Fe1—C5—C1	−46.2 (4)	C1—Fe1—C10—C6	77.4 (2)
C8—Fe1—C5—C1	163.2 (4)	C7—Fe1—C10—C6	−37.82 (18)
C10—Fe1—C5—C4	118.55 (19)	C8—Fe1—C10—C6	−81.41 (19)
C6—Fe1—C5—C4	161.75 (18)	C6—Fe1—C10—C11	−123.4 (3)
C3—Fe1—C5—C4	−37.82 (18)	C4—Fe1—C10—C11	37.8 (3)
C9—Fe1—C5—C4	76.3 (2)	C5—Fe1—C10—C11	−4.6 (3)
C2—Fe1—C5—C4	−81.6 (2)	C3—Fe1—C10—C11	70.9 (4)
C1—Fe1—C5—C4	−119.5 (3)	C9—Fe1—C10—C11	117.9 (3)
C7—Fe1—C5—C4	−165.7 (3)	C2—Fe1—C10—C11	−77.7 (4)
C8—Fe1—C5—C4	43.7 (5)	C1—Fe1—C10—C11	−46.0 (3)
C10—Fe1—C6—C7	−118.5 (3)	C7—Fe1—C10—C11	−161.2 (3)
C4—Fe1—C6—C7	−165.4 (3)	C8—Fe1—C10—C11	155.2 (3)
C5—Fe1—C6—C7	161.56 (19)	C9—C10—C11—O1	−5.8 (4)
C3—Fe1—C6—C7	45.6 (4)	C6—C10—C11—O1	−178.4 (3)
C9—Fe1—C6—C7	−80.3 (2)	Fe1—C10—C11—O1	−91.0 (3)
C2—Fe1—C6—C7	78.2 (2)	C9—C10—C11—C12	174.9 (3)
C1—Fe1—C6—C7	119.8 (2)	C6—C10—C11—C12	2.3 (4)
C8—Fe1—C6—C7	−36.84 (18)	Fe1—C10—C11—C12	89.7 (3)
C4—Fe1—C6—C10	−46.9 (4)	O1—C11—C12—Br1	5.7 (4)
C5—Fe1—C6—C10	−80.0 (2)	C10—C11—C12—Br1	−175.0 (2)
C3—Fe1—C6—C10	164.1 (3)		

### Hydrogen-bond geometry (Å, °)

**Table:**

Cg1 is the centroid of the C1–C5 ring.

**Table:**

*D*—H···*A*	*D*—H	H···*A*	*D*···*A*	*D*—H···*A*
C1—H1···O1^i^	1.00	2.49	3.327 (4)	140
C12—H12a···O1^i^	0.99	2.35	3.291 (4)	158
C12—H12b···Cg1^ii^	0.99	2.64	3.445 (3)	139

Symmetry codes: (i) −*x*+1, *y*+1/2, −*z*+3/2; (ii) *x*, −*y*+1/2, *z*−1/2.
